# An indirect enzyme-linked immunosorbent assay for the identification of antibodies to Senecavirus A in swine

**DOI:** 10.1186/s12917-017-0967-x

**Published:** 2017-02-15

**Authors:** Cheryl M. T. Dvorak, Zeynep Akkutay-Yoldar, Suzanne R. Stone, Steven J.P. Tousignant, Fabio A. Vannucci, Michael P. Murtaugh

**Affiliations:** 1 Department of Veterinary and Biomedical Sciences, University of Minnesota, 1971 Commonwealth Ave, St. Paul, MN 55108 USA; 2 Department of Virology, Ankara University, Faculty of Veterinary Medicine, Diskapi, 06110 Ankara, Turkey; 3 Swine Vet Center P.A., 1608 S. Minnesota Ave, St. Peter, MN 56082 USA; 4 Department of Veterinary Population Medicine, University of Minnesota, 1365 Gortner Ave, St. Paul, MN 55108 USA

**Keywords:** Seneca valley virus, SVV, Senecavirus A, SVA, Swine, ELISA, Veterinary diagnostics, Immunology

## Abstract

**Background:**

*Senecavirus A (SVA)*, a member of the family *Picornaviridae,* genus *Senecavirus*, is a recently identified single-stranded RNA virus closely related to members of the *Cardiovirus* genus. SVA was originally identified as a cell culture contaminant and was not associated with disease until 2007 when it was first observed in pigs with Idiopathic Vesicular Disease (IVD). Vesicular disease is sporadically observed in swine, is not debilitating, but is significant due to its resemblance to foreign animal diseases, such as foot-and-mouth disease (FMD), whose presence would be economically devastating to the United States. IVD disrupts swine production until foreign animal diseases can be ruled out. Identification and characterization of SVA as a cause of IVD will help to quickly rule out infection by foreign animal diseases.

**Methods:**

We have developed and characterized an indirect ELISA assay to specifically identify serum antibodies to SVA. Viral protein 1, 2 and 3 (VP1, VP2, VP3) were expressed, isolated, and purified from *E. coli* and used to coat plates for an indirect ELISA. Sera from pigs with and without IVD symptoms as well as a time course following animals from an infected farm, were analyzed to determine the antibody responses to VP1, VP2, and VP3.

**Results:**

Antibody responses to VP2 were higher than VP1 and VP3 and showed high affinity binding on an avidity ELISA. ROC analysis of the SVA VP2 ELISA showed a sensitivity of 94.2% and a specificity of 89.7%. Compared to IFA, the quantitative ELISA showed an 89% agreement in negative samples and positive samples from 4–60 days after appearance of clinical signs. Immune sera positive for FMDV, encephalomyocarditis virus, and porcine epidemic diarrhea virus antibodies did not cross-react.

**Conclusions:**

A simple ELISA based on detection of antibodies to SVA VP2 will help to differentially diagnose IVD due to SVA and rule out the presence of economically devastating foreign animal diseases.

## Background

*Senecavirus A (SVA)*, a member of the family *Picornaviridae,* genus *Senecavirus*, is a recently identified single-stranded RNA virus closely related to members of the *Cardiovirus* genus [1, 2]. SVA was originally identified as a cell culture contaminant and was not associated with disease until 2007 when it was first observed in pigs with Idiopathic Vesicular Disease (IVD) [2, 3]. Vesicular disease is sporadically observed in swine, is not debilitating, but is significant due to its resemblance to foreign animal diseases, such as foot-and-mouth disease (FMD), whose presence would be economically devastating to the United States [3, 4]. IVD disrupts swine production until foreign animal diseases can be ruled out. Identification and characterization of SVA as a cause of IVD will help to quickly rule out infection by foreign animal vesicular disease pathogens.

IVD in association with SVA has been observed recently in Canada, the United States, and Brazil, in the absence of other vesicular foreign animal diseases [3, 5, 6]. A quick test to diagnose SVA infection is necessary to help rule out infection by foreign animal diseases without prolonged disruption of animal movement. As of now, SVA infection is diagnosed by RT-PCR, a serum neutralizing assay, indirect fluorescent antibody test (IFA), or competitive enzyme-linked immunosorbent assay (cELISA) [6–9]. RT-PCR is a rapid method to determine if animals are acutely infected with virus or if vesicles contain virus, but a negative result cannot be used to rule out previous herd exposure since clinical signs of infection are usually resolved within 1–2 weeks [6, 10]. Presence of antibodies to SVA may indicate previous infection and possible presence of the virus in a herd. Although serum neutralization and IFA test for the presence of serum antibodies, ELISA is more rapid and convenient. A rapid, specific and sensitive assay for the detection of SVA-specific antibodies is needed. A cELISA for the detection of SVA antibodies is available, but requires an antibody competition between well characterized monoclonal antibodies and serum antibodies for binding to inactivated viral antigen [7]. An indirect ELISA only requires a purified antigen and so is not susceptible to mutations that change reactivity of the monoclonal antibody-binding epitope. An SVA VP1 ELISA has recently been used to examine antibody presence in sows and piglets naturally infected with SVA, however a comprehensive validation of this assay was not shown [11]. Although numerous ELISA kits used for the detection of viral antibodies are commercially available, an indirect ELISA kit is not yet commercially available for the detection of anti-SVA antibodies in pigs.

An optimized, well characterized, quick and inexpensive indirect ELISA for the detection of SVA antibodies as well as a thorough examination of antibodies and their levels over a time course following infection is needed. The aim of this study was to develop and characterize an indirect ELISA assay to identify serum antibodies to SVA as well as examine the kinetics of the presence and levels of SVA antibodies over a time course following infection. The SVA VP2 ELISA developed in this study can now be used to help differentially diagnose IVD due to SVA, helping to quickly rule out the presence of an economically devastating foreign animal disease.

## Methods

### Cloning, expression and purification of SVA VP1, VP2, and VP3 protein

The full length gene sequences for SVA VP1, VP2, and VP3 from strain 11-55910-3 (Genbank ID AGM16001) was optimized for expression in *E. coli*, synthesized (Integrated DNA Technologies, Inc., Coralville, IA), and cloned into a modified pET24b vector (Novagen, Madison, WI) [12] using the In-Fusion cloning kit (Clontech, Mountain View, CA) following the manufacturer’s directions. Gene sequences for VP1, VP2, and VP3 antigens were confirmed by sequencing. Protein expression and purification was performed as previously described [13]. Purity was analyzed by SDS-PAGE, stained using Imperial™ protein stain (ThermoFisher Scientific, Waltham, MA). Protein concentrations used to establish plate coating conditions were determined using the Quick-start Bradford protein assay following manufacturer’s instructions (Bio-Rad Laboratories, Inc., Hercules, CA) using a Bio-tek Epoch plate reader (BioTek Instruments, Inc., Winooski, VT).

### Serum samples

Recent and archived porcine serum samples that had been previously submitted to the University of Minnesota Veterinary Diagnostic Laboratory (UMN-VDL) for routine diagnostics or specific viral pathogen evaluation were obtained. The samples were provided for diagnostic purposes, not specifically for use in this study. In addition, positive serum samples were obtained from 34 sows clinically diagnosed with vesicular lesions and bled periodically over a 60-day period starting at the first observation of clinical signs (sampling at day 0, 4, 11,18, 25, 39, and 60) (*n* = 205). Serum samples that tested negative for SVA by PCR (*n* = 116) were obtained from sows and finishing pigs from various farms with no prior evidence of vesicular disease. These animals were assumed to be SVA antibody-negative and were treated as such in this study. Porcine epidemic diarrhea virus (PEDV) seropositive samples (*n* = 40) were archived samples from our laboratory and encephalomyocarditis virus (EMCV) seropositive samples were obtained from the UMN-VDL (*n* = 8). FMD seropositive samples (*n* = 21) were archived samples from Plum Island Animal Disease Center representing 8 different serotypes, 2 field samples, and ranged from 0 to 36 days post-infection/post-challenge. The FMDV samples were examined for cross-reactivity against the SVA VP1, VP2, and VP3 ELISA at Plum Island Animal Disease Center (PIADC) facilities following the ELISA protocol described here.

### Antibody detection by ELISA and IFA

Detection of antibodies to SVA VP1, VP2, and VP3 protein was performed by indirect ELISA as previously described [12, 13] on microtiter plates coated with either 500 ng or 200 ng of antigen per well or a combination of all 3 proteins at 100 ng each (300 ng total protein per well). Positive and negative control serum samples were run on each plate.

An avidity ELISA was performed following the ELISA protocol above, coating with 200 ng of VP2 antigen per well, and with the addition of a guanidine HCl wash step. Before secondary antibody was added, 1 M guanidine HCl in phosphate buffered saline (PBS) + 0.05% Tween-20, pH 7.4, was added to each well and incubated for 10 min. Plates were then washed as usual, secondary antibody was added, and the remainder of the ELISA protocol was performed as above. The avidity index was determined by dividing the optical density (OD) of the sample treated with guanidine by the OD of the sample without guanidine treatment (OD_Gn+_/OD_Gn-_).

The UMN-VDL performed a diagnostic IFA test to detect anti-SVA antibodies present in serum. Briefly, human lung cancer NCI-H1299 cells were inoculated with an SVA strain isolated in 2015 from an outbreak in the U.S. Infected cells were washed with PBS, fixed with acetone and incubated using two-fold dilutions of serum from 1:20 to 1:320 at 37 °C for 1 h. After fluorescein labeled goat anti-pig IgG diluted 1:50 in PBS was added into the wells and incubated at 37 °C for 1 h, the cells were observed under fluorescence microscopy. A positive signal at a sample dilution of 1:20 was considered suspect and a 1:40 or higher dilution was considered to be positive.

### Statistical methods

ELISA analysis, receiver-operator characteristics (ROC) analysis, and comparison to the IFA results were performed using GraphPad Prism software (Version 5.0a, GraphPad Software, Inc., La Jolla, CA).

## Results

### SVA protein expression and ELISA development

**Fig. 1:**
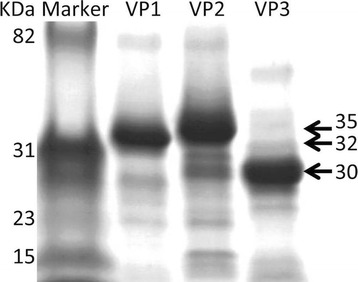
SVA VP1, VP2, and VP3 purified proteins. Purity of the proteins eluted from cobalt affinity columns were visualized by SDS-PAGE. Molecular weights are determined by comparison to kaleidoscope pre-stained standards (Bio-rad, Hercules, CA) run on the same gel

The SVA VP1, VP2, and VP3 proteins were cloned, expressed, and purified. Protein preparations were observed to be >90% pure (Fig. 1). ELISA plates were coated with 500 ng/well of VP1, VP2 or VP3 protein and samples were examined for anti-SVA antibody reactivity. SVA-negative samples from a neighboring farm (*n* = 28) were used to determine the cut-off values for negative (<OD_avg_ + 1SD) and positive (>OD_avg_ + 2SD) samples. Suspect positive samples occurred if the OD value fell between the negative and positive sample cut-off values.

**Fig. 2:**
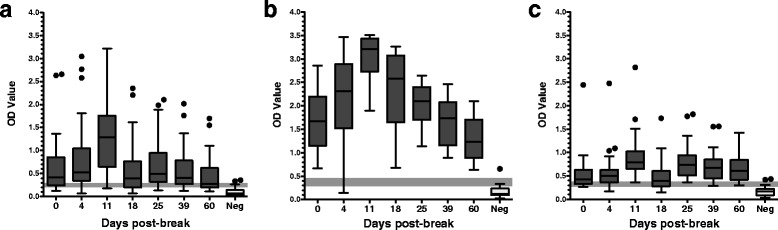
Time course of antibody responses to SVA VP1, VP2, and VP3. Serum samples collected from sows at the onset of clinical signs until 60 days later were tested in duplicate wells coated with 500 ng of each antigen. Negative control serum (Neg) was from a matched SVA-negative farm. Results are shown as a box whisker plot using the Tukey method for outliers for (**a**) VP1, (**b**) VP2, and (**c**) VP3 proteins. The suspect positive OD range is shown as a grey bar with negatives below and positives above the bar

The SVA ELISA was first evaluated using samples from a farm showing clinical signs, starting at the day clinical signs were first observed (Day 0) and sampled up to 60 days post-break (Days 4, 11, 18, 25, 39, 60). Comparison of antibody reactivity to VP1, VP2, or VP3 showed that VP2 was significantly different than VP1 and VP3 (*p* < 0.0001) giving the largest range of OD values and the biggest difference between positive and negative sample values (Fig. 2). VP1 showed some negative values at all time points and lower values overall, while VP3 showed limited immunoreactivity and discriminated poorly between positive and negative. A coating combination of 100 ng each of VP1, VP2, and VP3 together on the same samples shown in Fig. 2 gave results that were correlated with VP2 antigen alone (r^2^ = 0.927, *p* < 0.0001).

### SVA VP2 ELISA optimization and validation

Further optimization of VP2 ELISA sensitivity and specificity led to coating plates with 200 ng VP2 protein per well. ROC analysis was performed using 116 negative samples and 205 positive samples. At a positive/negative sample cut-off value of OD = 0.6, the area under the curve to differentiate positive from negative was 0.9622 with a *p* value <0.0001. Test sensitivity was 94.2% and specificity was 89.7%.

**Fig. 3:**
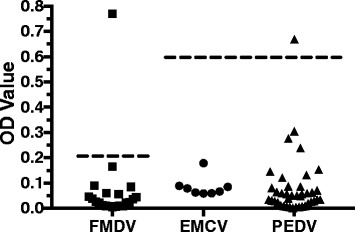
Cross-reactivity analysis of SVA VP2 ELISA to other virus seropositive samples. The SVA VP2 ELISA was performed on pig samples that were seropositive to other viruses (FMDV, EMCV, or PEDV), but whose SVA antibody status was unknown. The positive/negative cut-off values for EMCV and PEDV tested at the University of Minnesota, and FMDV tested at PIADC are shown by the dashed line

Cross-reactivity of the SVA VP2 ELISA was evaluated by testing samples that were seropositive for PEDV, a coronavirus, EMCV, a closely related picornavirus, and FMDV, a high-consequence animal picornavirus pathogen that causes vesicular lesions. We observed that 3% of PEDV-positive serum samples in a herd with no history of SVA exposure tested positive (ODavg = 0.081, SD = 0.119) (Fig. 3). All EMCV seropositive samples were negative for SVA antibodies (OD_avg_ = 0.085, SD = 0.040) (Fig. 3). FMD seropositive samples were examined for cross-reactivity on the VP1, VP2, and VP3 ELISAs. The FMDV ELISA was run at PIADC and the absorbance values (including positives and negatives) were lower than when the ELISA tests were run in our laboratory. Thus the cut-off values were adjusted based on the OD values of the positive and negative controls (Fig. 3). In the VP1 ELISA, 1 of the positive control samples was identified as suspect instead of positive and the VP3 ELISA identified 2 positive control samples as negative and 1 as suspect. For the FMDV-seropositive samples, 2 were identified as SVA VP1 positive and 4 as suspect, and no samples were positive for the VP3 ELISA. On the VP2 ELISA, 20 of the 21 (95.3%) samples tested negative and one (4.7%) sample tested positive. The FMDV positive sample that cross-reacted on the SVA VP2 ELISA was serotype A24. Notably, this sample also reacted with SVA VP1, suggesting that it was a true positive reaction and not cross-reactivity, since antibodies reacted against 2 SVA antigens.

### Avidity ELISA confirmation of reactivity

**Fig. 4:**
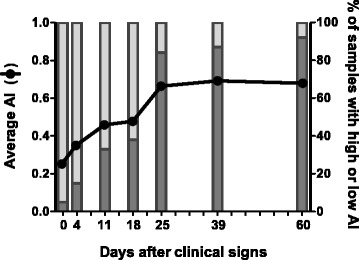
Avidity analysis on VP2 ELISA positive samples. The average avidity index (AI, *black line*) and percent of samples with low (<0.5, *light grey bar*) or high (>0.5, *dark grey bar*) AI are shown at each time point

Avidity, or functional affinity, measures the strength of a polyclonal antibody interaction with antigen, based on reduction in ELISA reactivity caused by incubation of the antigen-antibody complex with a denaturing agent. Figure 4 shows that the avidity index increased until 25 days after clinical signs were first observed. In addition, the proportion of samples with an avidity index >0.5 was greater than 80% at all times after 25 days (Fig. 4).

The avidity ELISA was also used to evaluate non-specific antibody reactivity against SVA VP2 protein that would give rise to false positive interpretations. The PEDV seropositive samples that cross-reacted with the SVA VP2 ELISA were evaluated by the avidity ELISA. These samples were clearly negative using the avidity ELISA (avidity index = 0.04). By contrast, the lowest avidity index in SVA seropositive samples from infected herds was 0.12 on the day clinical signs were first observed and the average avidity index of SVA seropositive samples was 0.52.

### Comparison of ELISA to IFA

**Fig. 5:**
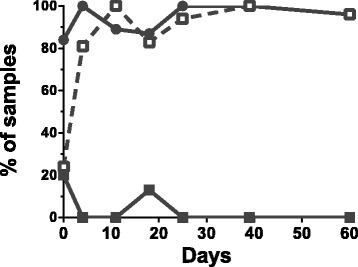
VP2 ELISA and IFA comparison. The percent of VP2 ELISA positive (*closed circles*) samples and the percent of IFA positive (*open squares*) and IFA suspect (*closed squares*) samples are shown over a 60 day time course

Comparison of negative samples and positive samples from the infection time course showed that ELISA and IFA were correlated (*n* = 231, *p* < 0.0001) (Fig. 5). The IFA has a specificity of 100% and a sensitivity of 90.3%. Agreement on negative samples was 89% and agreement from 4 to 60 days after appearance of clinical signs varied from 73 to 100%. However, on the day that clinical signs were first observed (Day 0), test agreement was 40%, which was due to samples testing ELISA positive, but IFA negative or suspect (Fig. 5). In cases of disagreement, ELISA was usually positive while IFA was negative or suspect. Three percent of total samples were ELISA negative, but IFA positive (days 11, 18, and 60).

## Discussion

Rapid diagnostic methods to test for SVA exposure in cases of vesicular disease are critical to the U.S. swine industry since animal movement, an essential aspect of modern swine production, can be halted if foot-and-mouth disease virus (FMDV) infection is suspected. The incidence of SVA has greatly increased since mid-2015, creating a compelling need for a simple, high-throughput test [10]. We have developed a sensitive and specific SVA ELISA for the detection and diagnosis of SVA-specific antibodies.

Surprisingly, VP2 antigen was substantially more immunogenic than VP1 and VP3 in the population as a whole, although occasional pigs were observed with high antibody levels to one or both of VP1 and VP3, as shown in Fig. 2. The sensitivity and specificity of the VP2 ELISA, 94.2 and 89.7%, respectively, indicate that it would be a reliable test to identify infected herds. The VP1 protein in picornaviruses is the most external and immunodominant [14] and has been used for numerous ELISA assays to detect picornavirus antibodies. However, many of the monoclonal antibody epitopes have been identified in VP2 and, because VP2 is more conserved than VP1, VP2 may be better able to detect across serotypes [15, 16]. Even though VP2 is conserved, cross-reactivity of the SVA VP2 ELISA to immune serum of the closely related picornavirus, EMCV, was not observed. Reactivity was observed in one (4.7%) FMDV seropositive sample. Since this serum sample, alone among 21 in total, also reacted with VP1, the most likely explanation is that the source animal was cryptically infected with SVA. The pig had been obtained from a commercial supplier and did not show clinical symptoms of disease, but its prior history was not known.

A﻿n avidity ELISA assesses the strength of antibody binding to antigen, thus denaturing weak interactions that are a common source of cross-reactivity and background noise [17]. Its use helps to increase assay specificity and to confirm suspect samples as positive or negative. In addition, an increase in avidity of the time course of an immune response can be used to monitor B lymphocyte maturation and antibody affinity increase that occurs during isotype switching and hypersomatic mutation in light and heavy chains [18]. We observed that the avidity index was low until about 3 weeks post-clinical signs, thus indicating that the humoral response was in an early stage and that infection was recent. It suggests that the avidity ELISA may be a useful indicator of outbreak initiation. Comparison of ELISA and IFA showed that ELISA also was more sensitive in diagnosing early infection. At later times (day 4–11 and later), ELISA and IFA were both able to identify SVA positive animals at similar rates, as shown in Fig. 5. Identification of SVA antibody-positive animals by SVA VP2 ELISA along with either the avidity ELISA or IFA as a confirmatory test in case of suspected false negatives, may be ideal for the immuno-diagnosis of SVA infection.

The kinetics of the antibody response to SVA have been recently examined. In a naturally infected farm ELISA-positive antibodies (anti-SVA VP1) were observed in pig serum at 1 week post-clinical signs in less than 15% of animals [11]. However, at 3 weeks post-clinical signs over 70% of animals were seropositive and by 6 weeks over 90% of animals were seropositive [11]. Experimental SVA infection of 9 week old pigs showed that all animals seroconverted by 15 days post-infection as determined by an indirect fluorescent antibody test [10]. In this study, we observed that SVA VP2 antibodies were present in 84% of animals when clinical signs were first observed and persisted for 60 days after clinical signs were first observed, though they were in decline from peak levels observed on day 11. In experimental infection of 9 week old pigs, vesicular lesions were first observed on the feet at 4 days, and nearly all pigs showed lesions at 5 days [10]. Since antibodies typically are not detected until 7 to 10 days after antigen exposure, it suggests that antibodies would not be present on the day clinical signs were first observed, as was observed by SVA IFA (only 24% of animals are seropositive by IFA on day 0) and by SVA VP1 ELISA, which was even less sensitive [11]. By contrast, the SVA VP2 ELISA is more sensitive, detecting antibodies in 84% of animals on the day of clinical signs.

## Conclusions

An indirect ELISA based on SVA VP2 can rapidly and reliably detect SVA antibodies present in swine. Identification of SVA infection as the cause of IVD can help to quickly rule out the presence of economically devastating foreign animal diseases in swine, enable producers to return to normal production after clinical signs have resolved, and make informed management decisions.
